# Moonshine as Medicine

**Published:** 1874-10

**Authors:** 


Moonshine as Medicine.—When the moon is nearly full many persons who lie down in the open air, exposed to its rays, suffer pains and œdema of the parts exposed, on the ensuing day, and sometimes they lose their reason when the moonlight is concentrated on the head; hence, no doubt the origin of the word "luney," or "lunatic," or moonstruck. I have treated several of these cases with the remedies recommended in the manuals, but never with rapid success till I had discussed the subject in my own mind awhile, and argued thus: If the moonlight *causes* the pain and œdema, there must be virtue in moonlight *to cure* it; so I exposed a glass half full of pure water to the direct and reflected light of the moon for three or four hours. At the end of this time I poured the water into a perfectly clean bottle, and shook it well for a moment or two. The next day I had a case of œdema of face and hands, with violent pains in the swollen parts, of a neuralgic nature, in a stout negro, of about thirty-five years of age, who had slept the previous night in the open air, exposed to the rays of *the full moon.* I gave him about two ounces of the prepared water from the bottle marked "Luna," with directions to take a spoonful every hour till relieved—this at 8 A.M.,; at 12 M., and after having taken three doses of the "*Luna,*" he was relieved of all pain, and at 4 P.M. *the œdema had entirely abated.* After such marked success, I treated several similar cases with *Luna,* with the same unvarying, quick relief. Reminding my friend, the Doctor, of these cases, I asked him if he had noticed whether Mrs. N. N.'s sufferings were aggravated at the time of full moon. This he had not noticed, but made a note of it to report at a future conference. About a month later, we met one evening, and he said that two days previously, (*the day after full moon*) Mrs. N. N. was taken suddenly with the most violent attack of metrorrhagia she had ever had, and her pains were most excruciating. Then, I replied, we must have found the key to the whole enigma; if I am right moonlight causes the disease, moonlight will cure it—give her *Luna.* A glass was prepared that night, and sent to her next morning, with directions to take a tablespoonful every hour till relieved. At the *third* dose the flow and pains ceased as if by magic. The ensuing month pains and flow presented themselves as usual, but *two* doses relieved entirely. A month later, pains and flooding again, but a single dose sufficed this time, and the month following, menstruation was normal—no pains or excess of flow. Thus this patient, after two years' suffering and agony, was restored to perfect health. Three years afterwards there had been no relapse. After her cure, she remembered distinctly that every time she sat exposed to the moonlight at the full of the moon, her sufferings were in every way aggravated; when she kept in the house at this epoch, she suffered much less—now she sits exposed to the moonlight for three or four hours with impunity.

We prepared *Luna,* and protentized it up to the 13th potency —a powder of which I have furnished to Dr. Samuel Swan, 13 West Thirty-eighth street, New York, and it will soon be potentized up to the *cm* potency by his new potentizing machine, just finished. I have used it for *all cases of abnormal menstrual troubles which are aggravated at the period of full moon*, at the 6th cent. potency, without having, as yet, to record a case of failure. Relating the preceding case to Dr. Swan, in December, 1872, he prepared some Sac. lac. by exposure to the concentrated rays of the sun, and has had this potentized by Dr. Finke up to the *cm*, and with different high potencies has cured severel cases of headache where patients could remember having suffered at any previous time *by exposure to the sun*. I believe that in such cases, which do not yield readily to other remedies, it will prove a specific; and, as such, of great value in our M. Medica. Dr. Fincke has potentized *Luna* up to the *cm* potency; but this is from the moonlight in our climate, which, I think, may possibly be less powerful than the preparation I brought from South America; but as yet I have not had any opportunity of testing it, so as to institute any comparison between the effects developed by the one and those developed by the other.—*North American Journal of Homœopathy.*

